# Supplementary material for: Propranolol, chlorpromazine and diclofenac restore susceptibility of extensively drug-resistant (XDR)-Acinetobacter baumannii to fluoroquinolones
Source: PLoS One. 2020 Aug 26;15(8):e0238195. doi: 10.1371/journal.pone.0238195 (PMC7449414; doi:10.1371/journal.pone.0238195)
Supplement: S1 Fig — (PDF) [file pone.0238195.s001.pdf]

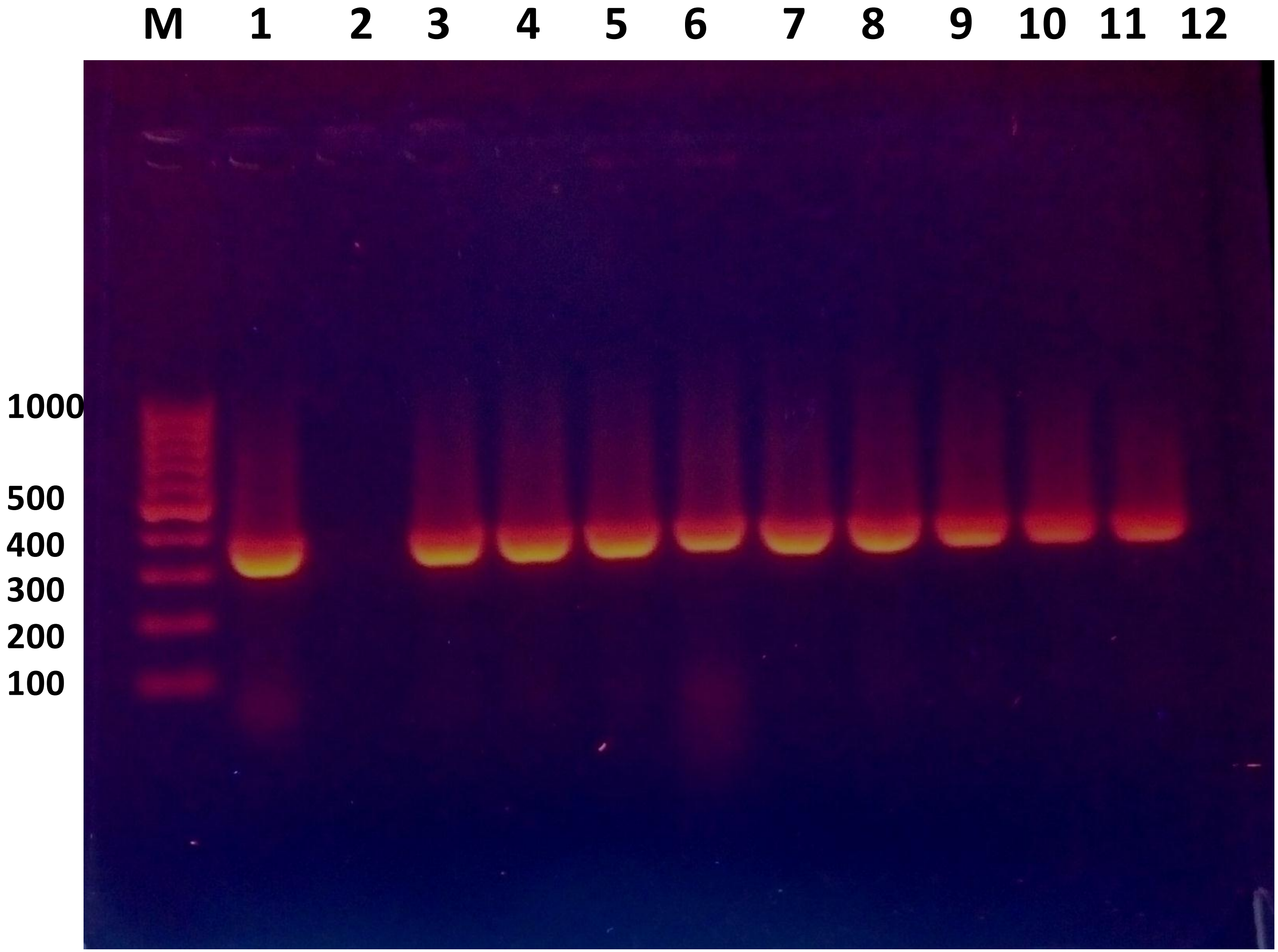

Fig 2 (raw): Agarose gel electrophoresis of PCR amplification of *bla*$_{OXA-51}$ like gene in some *A. baumannii* clinical isolates, lane M, a gene Ruler 100 bp ladder; lane 1, a positive control; lane 2, a negative control; lanes 3 to 11, positive results with an expected size of 353 bp; lane 12, negative results
