# Supplementary material for: Propranolol, chlorpromazine and diclofenac restore susceptibility of extensively drug-resistant (XDR)-Acinetobacter baumannii to fluoroquinolones
Source: PLoS One. 2020 Aug 26;15(8):e0238195. doi: 10.1371/journal.pone.0238195 (PMC7449414; doi:10.1371/journal.pone.0238195)
Supplement: Attachment — Submitted filename: Response to Reviewers.docx [file pone.0238195.s003.docx]

**Author's Response to Reviewer's comments**

On the behalf of all authors, we would like to thank the reviewers for their valuable comments and suggestions that will improve and add to this manuscript. Corrections are highlighted in yellow color and all have been included in the revised manuscript.

**Abstract :
Title: Please change extensive to extensively. The authors probably misinterpret my suggestion that they should qualify “extensively drug resistant” in the keyword, hence they went ahead to change the phrase throughout the manuscript to “extensive”. What I meant is that they should add what is extensively resistant to complete the keyword, in this case A. baumannii.**

Author response:

Thank you for the reviewer's comment. We updated the title and updated the key words

**Line 27: change “of” to “caused by”**

Author response:

Thank you for the reviewer's comment. done (page2, line 27)

**Line 29-30: “....and evaluating the various combinations to combat ....” evaluating the various combination of what? In addition, authors should please change “evaluating” to “evaluate”**

Author response:

Thank you for your valuable comment. We updated to **“** evaluate the various combinations of ciprofloxacin or levofloxacin with antimicrobial agents and non-antimicrobial agents to combat antimicrobial resistance of XDR *A.* *baumannii***”**.(page 2; lines 30-31)

**Line 32: Delete “a total of” before 1600**

Author response:

Thank you for the reviewer's comment. We deleted “a total of”. (page 2, line 33)

**Line 36: Change to “high percentage of bacterial resistance to 19.....”**

Author response:

Thank you for your valuable comment. We updated to **“**a high percentage of bacterial resistance to 19”(page 2, line 37)

**Line 42: Please include CPZ, PR and DIC among drugs tested in combination with CIP and LEV**

Author response:

Thank you for the reviewer's comment. this part was updated since CPZ, PR, and DIC are among drugs that were tested according to MIC decrease factor (MDF) protocol however the antibiotic combinations were evaluated according to standard protocol of fractional inhibitory concentrations (ƩFICs) (line 43). So we put them in aseprate senctence for more clarifications (page2, lines 47-49)

**Lines 43-44: Please change “have been tested” to “was tested” and delete “including 2 PDR and 32 XDR**

Author response:

Thank you for your valuable comment. We updated the sentence (page2, line 43).

**Lines 51-53: Delete the sentence starting with in conclusion, it is not necessary.**

Author response:

Thank you for the reviewer's comment. We deleted the sentence.

**Line 55: Delete “ The obtained results are still promising in vitro study” and start the sentence with However.......**

Author response:

Thank you for your valuable comment. We updated the sentence (page 3, lines 51-53).

**Line 57: Please delete “a life-threatening pathogen”.**

Author response:

Thank you for the reviewer's comment. We deleted the sentence (page 3, line 55).

**Line 58: Delete “extensive drug-resistant” it is more like a repetition of the first keyword XDR-A. baumannii**

Author response:

Thank you for the reviewer's comment. We deleted the sentence.

**Selection of isolates for inclusion in the drug combination experiment
I am still not comfortable with the criteria used by the authors in selecting isolates for the drug combination experiment. According to them, they selected the 34 isolates based on drug susceptibility profile. While this is scientific enough, it remains a curiosity how they selected which among the 68 XDR isolates to include since according to them (Lines 230-231) all the 68 XDR isolates shared exactly the same susceptibility profile i.e. resistance to all antibiotics except colistin. Again, one wonders why representatives of the MDR strains are not included in the study as a 3rd category since the work is about antibiotic resistant A. baumannii?**

**Author response:**

Thank you for your valuable comment. Yes, we selected the 34 isolates based on the drug susceptibility profile. The clinical isolates which were included, (2 PDR and 32 XDR) The selected 32 showed the highest resistant patterns (all were resistant to 19 teste antibiotics except colistin) while other resistant profiles were those which were susceptible to 2 drugs of the 13 drugs tested. So, we selected the isolates of the highest resistance patterns (life-threatening isolates from medical pont of view). In addition , the the selected isolates non-clonal. Future studies will be conducted for testing these combinations on large number of isolates and to evaluated in vivo using appropriate animal model.

**Introduction
I appreciate that the authors have updates this section of the manuscript with new information. However, I still have concerns about the writing style and presentation which needed considerable improvement.**

**Author response:**

Thank you for your valuable comment. The whole manuscript has been revised thoroughly for any grammar or any spelling or amy possible typing errors.

**Line 62: insert the word “bacteria” after aerobic and after common in line 65**

Author response:

Thank you for the reviewer's comment. added . (page 3, line 59)

**Line 157: Delete “against” after isolates**

Author response:

Thank you for the reviewer's comment. deleted . (page 7, line 151)

**Lines 159-160: Delete “of”**

Author response:

Thank you for the reviewer's comment. We deleted "of". (page 7, line 156)

**Line 180: Change “a MIC” to “the MIC”**

Author response:

Thank you for the reviewer's comment. We changed it. (page 8, line 170)

**Line 185: did the authors mean to write two instead of tow**

Author response:

Thank you for the reviewer's comment. Yes, we changed it (page 8, line 175).

**Line 193: ....antagonistic when ΣFIC is ˃ than what?**

Author response:

Thank you for the reviewer comment. “when ƩFIC is > 4”, we changed it (page 8, line 183).

**Line 196: The authors should mention the different concentrations of each non-antibiotic drugs tested may be as supplementary information. While they may think this is not very important, it does provide a valuable guide for others who may want to repeat this experiment.**

Author response:

We added the different concentrations of non-antimicrobial agents (page 8, lines 186-191)..

**Line 207: Range should be from low to high and not otherwise as presented here.**

Author response: corrected (page 9, line 195)

**Lines 214-216: In view of the fact that the target organism in this study is a non-lactose fermeter, I wonder why the authors are mentioning the isolation of lactose fermenting colonies in this section. I will rather they delete all reference to lactose fermenters as it has no relevance in this work.**

Author response:

Thank you for the reviewer's comment. we updated the sentence. (page 9, line 203)

**Lines 223-224: The authors claimed that the lowest resistance was exhibited towards doxycycline at 57% followed by colistin at 5%. 57% could not be lower than 5%.**

Author response:

Thank you for the reviewer comment. we updated the sentence (page 9, line 211)

**Discussion
The discussion section, much like the whole manuscript still needs considerable improvement in writing style. In addition, much of this section is just a combination of results with previous studies without the authors providing the much needed depth of insight into their results. They also devoted a lot of space to discussion on isolation, antibiotic susceptibility and genotyping of the isolates through ERIC-PCR at the detriment of the real focus of their research which is the evaluation of drug combination in the control of resistant A. baumannii. The resultant effect of this is an overly long discussion section.**

Author response:

Thank you for your valuable comment. We revised the discussion and we discussed the results according to the available literature however, there were only few studies on fluoroquinolone combination with antimicrobial agents are available. We revised the dicsusion and we did our best to make it more concise about the drug combinations.

**Line 321: The authors should please correct the doxycycline susceptibility data to 43% since they earlier mentioned that resistance to doxyxcycline was 57%.**

Author response:

Thank you for the reviewer's comment. we changed it. (page 24, line 320)

**Lines 377-384: This explanation as presently offered does not help make a case for the low number of isolates included in this study. Claiming that the experiment took a lot of time, effort and resources and was carried out in triplicates is at best not tenable. In my own opinion, these are essential ingredients one may not be able to avoid in the business of science. Pointing out that the study provided baseline information upon which a wider study could be based would have been a better alternative.**

Author response:

Thank you for the reviewer's comment. this part of in the discussion have been updated (page 27, lines, 380-384)

**Line 397: Authors mentioned that their results did not agree previous study in north Egypt. They should please cite that study for reference here.**

Author response:

Citation has been include.(page 27, line 380)

**Similarly in Lines 404-405 authors claimed their results agree with previous study in upper Egypt, they should cite that study also for reference.**

Author response:

Citation has been included (page 27, line 389)

**Lines 414-416: An incomplete sentence**

Author response:

Corrected (page 27, line 383-384)

**I suppose the values in bracket in Tables 2 and 3 are the baseline MICs of CIP and LEV? If this is so, this should be indicated in the legends to the table.**

Author response:

The baselines od MICs of CIP and LEV are provided in table 1. In tables, 2 and 3 are the MICs of the two tested antibiotics after their combinations (this was clarified in the legends of tables 2 and 3).

**Figures Legend- I think the authors should check again the journal requirement about figures is that the figure caption and legend be inserted immediately after the paragraph where the figure was first mentioned.**

Author response:

The legends of all figures are provided at the end of the manuscript according to the journal requirement.

**Figure 3. The authors should please correct the value for CT, 95% would not have been that small on the chart.**

Author response:

It was provided as % resistance (5% resistance) = 95% sensetive

**Reviewer #2:**

**This manuscript has been improved, and has very interesting results, but:
1. The authors should revise again the format of the manuscript. Some spaces are missing, incorrect used punctuation marks, missing words and repetition of others (clearly from copy and paste some sentences), as well as words misspelling.**

Author response:

Thank you for the reviewer's comment. Thank you for your valuable comment. The whole manuscript has been revised thoroughly for any grammar or any spelling or typing errors

.

**2. It is not needed to repeat the meaning of the abbreviations along with the text as far they explained the first time.**

Author response:

Thank you for the reviewer's comment. all have been revised and corrected

**3. The term “our findings” is not properly used.**

Author response:

Thank you for the reviewer's comment. We replaced “our findings” by “present study”, and “in this study”. (page 24; line 320, apge 25, line 329)

**4. Line 132. The authors repeat the amount of DNA added to the PCR, this information was provided in line 130.**

Author response:

Thank you for the reviewer's comment. Information was provided in line 130, represent final concentration of DNA (100 ng of genomic DNA,), while Information was provided in line 132, represent the volume of DNA was added to PCR mixture (and 1 µl template DNA). However, we deleted it (containing 100-200 ng of chromosomal DNA) (page 6, line 128).

**5. Line 135-136. The sentence implies that they used the UV illuminator before doing the electrophoresis gel.**

Author response:

Corrected to " PCR products were analyzed using agarose gel electrophoresis [23]. " (page 6, line 131)

**6. Line 197, 387, 433. “&” is not appropriate for a text, should be changed for “and”, “as well as” or other synonym chosen by the authors.**

Author response:

Thank you for your valuable comment. we changed it to “and”in whole manuscript

**7. Tables 2 and 3. Authors should add to the table legend the meaning of the numbers between brackets, and use the same names in the table and the legend (CIP-AMP or AMP-CIP).**

Author response:

There are the MIC of the two antibiotics after their cobmibations. (was clarified and included in the legends of tables 2 and 3)

**8. Table 2. There is no A in the table, so there is no point in explaining it in the legend.**

Author response:

Deleted since we did not have antagonism (deleted)

**9. Table 4. FIC meaning missing.**

Author response:

Thank you for the reviewer's comment. FIC, fractional inhibitory concentration (We added it in the legend of table 4)

**10. Line 299. I think the authors may want to say bacteremia or blood infection.**

Author response:

Thank you for your valuable comment. We changed it to a blood infection (page 24, line 298).

**11. Lines 397-406. This paragraph was not clearly written, some sentences are incomplete.**

Author response:

Thank you for your valuable comment. We changed it It has been amended according to reviewer 1 by added references 9 and 10.

**12. Lines 426-436. Sentences incomplete. Paragraph unintelligible, clearly a result of copy and paste sentences.**

Author response:

Revised and corrected.

**Dear Dr. Monica Cartelle Gestal, Ph.D., Academic Editor, PLOS ONE,**

On behalf of all authors who participated in writing this manuscript, we would like to express our deepest gratitude to the reviewers for their valuable comments that enriched the revising process. All comments and recommendations by reviewers were done and the whole manuscript was revised thoroughly for any typing or grammatical mistakes. On behalf of all co-authors, we appreciate your help, effort and kind support.

Kind regards

Corresponding author,

Prof Dr. Khaled Aboshanab (Ph.D.)
